# Predictors of the pulsatility index in the middle cerebral artery of acute stroke patients

**DOI:** 10.1038/s41598-020-74056-2

**Published:** 2020-10-13

**Authors:** Olivier Bill, Dimitris Lambrou, Guillermo Toledo Sotomayor, Ivo Meyer, Patrik Michel, Tiago Moreira, Julien Niederhauser, Lorenz Hirt

**Affiliations:** 1 Stroke Unit, GHOL, Nyon Site, Nyon, Switzerland; 2 Stroke Center, Neurology Service, Department of Clinical Neurosciences, Lausanne University Hospital (CHUV) and University of Lausanne, Bugnon 46, 1011 Lausanne, Switzerland; 3 Department of Neurology, Karolinska Stroke Research Unit, Karolinska University Hospital-Solna, Stockholm, Sweden

**Keywords:** Neurology, Predictive markers, Neurological manifestations, Arterial stiffening, Carotid artery disease, Thromboembolism, Cerebrovascular disorders, Stroke

## Abstract

Cervical and transcranial Doppler (TCD) are widely used as non-invasive methods in the evaluation of acute ischemic stroke (AIS) patients. High-grade carotid artery stenosis induces haemodynamic changes such as collateral flow and a so-called post-stenotic flow pattern of the middle cerebral artery (MCA), which appears flattened, with a reduction of the velocity difference between systole and diastole. We studied the influence of carotid artery stenosis and other variables on the flow pattern in the MCA using the pulsatility index (PI), a quantitative TCD parameter reflecting the flow spectrum in a large of cohort AIS patients. We performed ultrasound examinations of 1825 AIS patients at the CHUV from October 2004 to December 2014. We extracted patient characteristics from the ASTRAL registry. Carotid stenosis severity was classified as < 50%, 50–70%, 70–90% and > 90%, or occlusion, according to Doppler velocity criteria. We first determined variables associated with stenosis grade. Then we performed a multivariate analysis after adjusting for baseline differences, using MCA PI as dependent variable. Carotid stenosis > 70% (− 0.07) and carotid stenosis > 90%, or occlusion (− 0.14) and left side (− 0.02) are associated with lower MCA PI values. Age (+0.006 PI units per decade), diabetes (+0.07), acute ischemic changes on initial CT (+0.03) and severe plaque morphology (+0.18) are associated with higher MCA PI values. We found a number of clinical and radiological conditions that significantly influence the PI of the MCA, including high-grade ipsilateral carotid stenosis in AIS patients. We provide for the first time a quantitative evaluation of the effect of these influencing factors from a large cohort of AIS patients.

## Introduction

Transcranial Doppler (TCD) is used for the evaluation of acute ischemic stroke (AIS) patients. It is a valuable adjunct to CT angiography (CTA), providing complementary information such as presence of collateral flow, steal syndromes and real-time detection of microemboli^1–3^. Furthermore, it plays an important role in the early follow-up after an AIS as it can detect arterial recanalization or persistent occlusion at 24 h with an accuracy comparable to that of digital subtraction angiography (DSA), computed tomography angiography (CTA) or magnetic resonance angiography (MRA)^4,5^. Vessel patency is associated with outcome status^6–8^ in the hyperacute phase and early after recanalization therapy^9,10^.

Pulsatility index (PI) is a rheological TCD parameter that reflects distal cerebrovascular flow resistance^11,12^. Recently, it was pointed out that PI not only mirrors the cerebrovascular resistance but is also influenced by the cerebral perfusion pressure, the cerebral arterial compliance and heart rate (De Riva et al., Neurocrit Care (2012) 17:58–66). PI is independent of the probe angle to the vessel, the carrier frequency and the velocity of sound in tissues^1,13^. The cerebrovascular resistance is influenced by intracranial pressure, as the intracranial pressure increases, the diastolic flow velocity decreases due to an increased distal resistance resulting in increased pulsatility^12^. The PI has been used for non-invasive intracranial pressure estimations although its interpretation needs caution^14^.

The PI is also used for recanalization scores in acute ischemic stroke to assess residual stenosis after recanalization interventions. Additionally, it is used in the Thrombolysis in Brain Ischemia (TIBI) score, applying TCD to assess arterial patency after AIS treatment. The TIBI score correlates with the clinical outcome and mortality after IV thrombolysis^15,16^ and mechanical thrombectomy^17^. Furthermore, it is correlated with an adapted Thrombolysis In Myocardial Infarction (TIMI) score for intracranial arteries^16,18^.

Results from animal studies suggest that PI increases if stenosis is distal to the site of insonation and decreases if stenosis is found proximal to the examination area^11^. So far, the influence of patient characteristics and specifically, of concomitant pre-cerebral stenosis on intracranial TCD parameters has only been scarcely studied. One study^19^ used TCD to assess the arterial status after TPA therapy in ischemic stroke in sixty stroke patients. This study proved that TCD can identify persistent occlusion and provides some information on MCA PI values in the setting of ischemic stroke and carotid stenosis or occlusion. We hypothesised that the pre-cerebral stenosis or occlusion (and possibly other factors modifying cerebrovascular resistance) influence MCA PI measures in AIS patients and tested this hypothesis on a large number of patients.

### Methods

The present study is a retrospective single-centre analysis of ultrasound examinations performed on consecutive, prospectively examined acute (< 24 h) anterior circulation ischemic stroke patients. Patients were referred during their hospital stay to the cerebrovascular ultrasound laboratory at the University Hospital of Lausanne (CHUV) from October 14th, 2004 to December 31st, 2014. We extracted Doppler data from the Lausanne Doppler Registry (LaDoRe), including Peak Systolic Velocity (PSV), End-Diastolic Velocity (EDV) and Pulsatility Index (PI) of the ICA, MCA and ACA. We focused on the PI values in the MCA ipsilateral to the carotid stenosis. We analysed each carotid side separately and compared the results to the MCA PI of carotids without stenosis. We performed Doppler examinations within 7 days of patient admission and considered only patients with anterior circulation strokes. We used three distinct measures from different depths for each MCA and for each patient and then used the mean per patient of the same vessel to calculate the PI.

Patients were divided into 4 groups: NO for no stenosis corresponding to patients with a stenosis grade in the ICA of < 50%, LO for low-grade stenosis for patients with stenosis between 50 and 70%, HI for high-grade stenosis for stenosis between > 70 and 90%, and group SO for sub-occluded/occluded in patients with stenosis of > 90% or occlusion.

We graded stenosis of the ICA using velocity criteria for stenosis < 90% and combined velocity criteria and collateral flow assessment through ophthalmic and anterior cerebral arteries for stenosis > 90%^20^. We also assessed the burden of overall atherosclerosis in the exam according to the Mannheim plaque consensus^21^ internal criteria and placed into 4 categories: none, light (plaques are homogenous or hyperechogenous), moderate (heterogeneous multiple plaques, hypoechogenous plaques) and severe (extended plaques with ulcerations or haemorrhage).

Patients were excluded from the analysis for the following reasons: under 18 years of age, critical data lacking in patient’s health record, incomplete neurosonological exam, non-interpretable exam due to technical difficulties and absent target vessel velocity data. Bilateral carotid stenosis and pre-existing or distal tandem intracranial stenosis patients, as well as patients with stented carotids were also excluded.

We extracted clinical, demographic and biological data from the ASTRAL registry^22^), which includes only AIS patients within 24 h of last proof of good health. The data were then merged with the Doppler data. The use of all data was approved by the ethics committee of the Canton de Vaud, sub commission III, project N°34/15.

Initially we performed descriptive univariate analysis to assess how the baseline ultrasound distribution for NO, LO, HI and SO groups was associated with the covariates of interest to better assess the profile of the population of interest.

In a second analysis, we performed a multivariate analysis with MCA PI as the response variable to investigate significant relationships with preselected covariates. Prior to multivariate analysis, we performed imputation of missing data using the chains equations method. In this process, five imputed datasets were generated. Each dataset was analysed separately and the significant associations of the response with the covariates were determined using stepwise methods. Finally, the analyses of the five datasets were appropriately combined to derive the conclusions of our study. In all multivariate analyses, allowance was made for the possible stochastic association of MCA PI measurements on the same patient, using a compound symmetric error structure.

For continuous and ordinal variables, median and interquartile range (IqR) values were calculated. For categorical variables, number and percent were used as summary measures.

All analyses were performed using the R statistical package (version 3.5.3).

### Results

13,719 Doppler examinations were available in the LaDoRe that included 1821 patients that had1825 anterior circulation AISs. Baseline patient profiles (described in Table 1) were as follows: admission NIH stroke scale (NIHSS) 7 (IqR 11), age 73.8 years (IqR = 18.9), 1686 (56.9%) men, 1662 patients (60%) were independent at 3 months (modified Rankin scale, mRS 0–2). Strokes were classified into subtypes according to TOAST criteria: there were 958 (33.6%) cardioembolic strokes, including 884 (28.5%) cases of atrial fibrillation; 456 (16%) suffered from atherosclerosis, which corresponds to the overall ASTRAL population. One hundred and forty-three (5%) had watershed territory strokes and 207 (7%) peripheral arterial disease. 1152 (41.7%) had an acute ischemic lesion on admission CT, 1470 (63.1%) had a significant arterial stenosis or occlusion in the ischemic territory on acute Angio CT.

**Table 1:** Univariate analysis of the distribution of variables associated with the different stenosis groups.

Variable	Stenosis grade group (categorical: n (%)—continuous: median (IQR))							
	NO (N = 2556)	LO (N = 165)	HI (N = 67)	SO (N = 180)	Total (N = 2968)	OR	95% CI	*p* value
Age	74.0 (19.1)	76.4 (15.2)	75.4 (14.9)	66.0 (22.0)	73.8 (18.9)	1.00	0.99–1.00	29.2%
**Sex**								
Males	1410/2554 (55.2%)	106/164 (64.6%)	50/67 (74.6%)	120/179 (67.0%)	1686/2964 (56.9%)			< 1%
Females	1144/2554 (44.8%)	58/164 (35.4%)	17/67 (25.4%)	59/179 (33.0%)	1278/2964 (43.1%)	0.60	0.48–0.74	
**Stroke arterial territory**								
Anterior	1995/2484 (80.3%)	138/160 (86.2%)	59/64 (92.2%)	169/176 (96.0%)	2361/2884 (81.9%)			< 1%
Posterior	306/2484 (12.3%)	14/160 (8.8%)	2/64 (3.1%)	5/176 (2.8%)	327/2884 (11.3%)	0.37	0.23–0.56	
Simultaneous	45/2484 (1.8%)	4/160 (2.5%)	2/64 (3.1%)	2/176 (1.1%)	53/2884 (1.8%)	0.94	0.41–1.88	
Watershed	138/2484 (5.6%)	4/160 (2.5%)	1/64 (1.6%)	0/176 (0.0%)	143/2884 (5.0%)	0.19	0.07–0.43	
**Stroke mechanism**								
Atherosclerosis	265/2449 (10.8%)	73/157 (46.5%)	39/64 (60.9%)	79/179 (44.1%)	456/2849 (16.0%)			< 1%
Cardioembolic	914/2449 (37.3%)	17/157 (10.8%)	8/64 (12.5%)	19/179 (10.6%)	958/2849 (33.6%)	0.07	0.05–0.10	
Microangiopathy	283/2449 (11.6%)	16/157 (10.2%)	1/64 (1.6%)	0/179 (0.0%)	300/2849 (10.5%)	0.08	0.05–0.14	
Dissection	101/2449 (4.1%)	6/157 (3.8%)	0/64 (0.0%)	36/179 (20.1%)	143/2849 (5.0%)	0.71	0.47–1.06	
Unknown	679/2449 (27.7%)	16/157 (10.2%)	3/64 (4.7%)	13/179 (7.3%)	711/2849 (25.0%)	0.07	0.04–0.10	
Other	101/2449 (4.1%)	4/157 (2.5%)	1/64 (1.6%)	6/179 (3.4%)	112/2849 (3.9%)	0.16	0.08–0.29	
Multiple	106/2449 (4.3%)	25/157 (15.9%)	12/64 (18.8%)	26/179 (14.5%)	169/2849 (5.9%)	0.84	0.59–1.18	
**Risk factors**								
Hypertension	1862/2550 (73.0%)	137/165 (83.0%)	56/67 (83.6%)	116/180 (64.4%)	2171/2962 (73.3%)	1.07	0.84–1.36	59.4%
Diabetes	534/2553 (20.9%)	42/164 (25.6%)	15/67 (22.4%)	36/180 (20.0%)	627/2964 (21.2%)	1.09	0.85–1.39	49.7%
Hypercholesterolemia	1952/2540 (76.9%)	140/165 (84.8%)	56/67 (83.6%)	137/180 (76.1%)	2285/2952 (77.4%)	1.24	0.96–1.62	9.7%
Atrial fibrillation	758/2547 (29.8%)	32/165 (19.4%)	15/67 (22.4%)	39/179 (21.8%)	844/2958 (28.5%)	0.63	0.49–0.81	< 1%
Peripheral arterial disease	152/2533 (6.0%)	24/163 (14.7%)	15/67 (22.4%)	16/178 (9.0%)	207/2941 (7.0%)	2.31	1.66–3.17	< 1%
**Stroke evolution**								
NIHSS at admission	7.0 (11.0)	6.0 (10.0)	6.0 (6.5)	12.0 (12.5)	7.0 (11.0)	1.03	1.01–1.04	< 1%
Patient independent at 3 months (mRs = 0–2)	1460/2399 (60.9%)	88/148 (59.5%)	37/60 (61.7%)	77/168 (45.8%)	1662/2775 (59.9%)	0.73	0.59–0.91	< 1%
**Imaging characteristics**								
Any ischemic lesion on acute CT imaging	964/2373 (40.6%)	61/153 (39.9%)	28/63 (44.4%)	99/173 (57.2%)	1152/2762 (41.7%)	1.40	1.13–1.74	< 1%
Significant atherosclerosis on acute imaging	1408/1991 (70.7%)	113/125 (90.4%)	50/50 (100.0%)	105/154 (68.2%)	1676/2320 (72.2%)	1.73	1.30–2.35	< 1%
Significant arterial stenosis or occlusion in ischemic territory on acute Angio CT	1169/1999 (58.5%)	104/125 (83.2%)	47/53 (88.7%)	150/152 (98.7%)	1470/2329 (63.1%)	7.53	5.19–11.37	< 1%
Haemorrhagic transformation according to ECASS definition on subacute imaging	34/2403 (1.4%)	2/158 (1.3%)	0/65 (0.0%)	4/175 (2.3%)	40/2801 (1.4%)	1.10	0.41–2.45	83.3%
Delay until first Doppler exam (d)	2 (2)	1 (1)	1 (1)	1 (1)	1 (2)	0.84	0.77–0.91	< 1%
Right MCA PI	1.05 (0.3)	1.12 (0.3)	1.10 (0.4)	0.83 (0.3)	1.04 (0.3)	0.45	0.26–0.78	< 1%
Left MCA PI								
IMT R common carotid artery	0.9 (0.3)	0.9 (0.4)	0.9 (0.4)	0.9 (0.3)	0.9 (0.3)	1.61	1.11–2.30	1.2%
IMT left common carotid artery								
**Overall plaque score**								
None	634/2539 (25.0%)	23/163 (14.1%)	4/66 (6.1%)	35/162 (21.6%)	696/2930 (23.8%)			< 1%
Light atherosclerosis	983/2539 (38.7%)	38/163 (23.3%)	17/66 (25.8%)	50/162 (30.9%)	1088/2930 (37.1%)	1.09	0.79–1.52	
Moderate atherosclerosis	882/2539 (34.7%)	95/163 (58.3%)	43/66 (65.2%)	72/162 (44.4%)	1092/2930 (37.3%)	2.36	1.76–3.21	
Severe atherosclerosis	40/2539 (1.6%)	7/163 (4.3%)	2/66 (3.0%)	5/162 (3.1%)	54/2930 (1.8%)	3.39	1.71–6.38	

Regarding Doppler characteristics, 2985 carotids were examined (1491 left carotids and 1494 right carotids). In 810 cases, the corresponding MCA examination was not available and the analyses were therefore performed on 2175 carotid/MCA pairs. Seventeen patients had either bilateral carotid sub-occlusion or occlusion or occlusion of one ICA combined with a significant stenosis (> 70% or above) on the contralateral side and thus were excluded. The median number of days before Doppler examination was one and the median intima-media thickness (IMT) was 0.9. In 1676 (72.2% of the cases), significant arteriosclerosis was observed on acute imaging, either intra- or extra-cranially. Regarding pre-cerebral plaque severity, 696 (23.8%) had no plaques, 1088 (37.1%) had a light atherosclerosis, 1092 (37.3%) had a moderate atherosclerosis, and 54 (1.8%) had a severe atherosclerosis. 41 carotids were lacking information on the plaque morphology.

**Figure 1:**
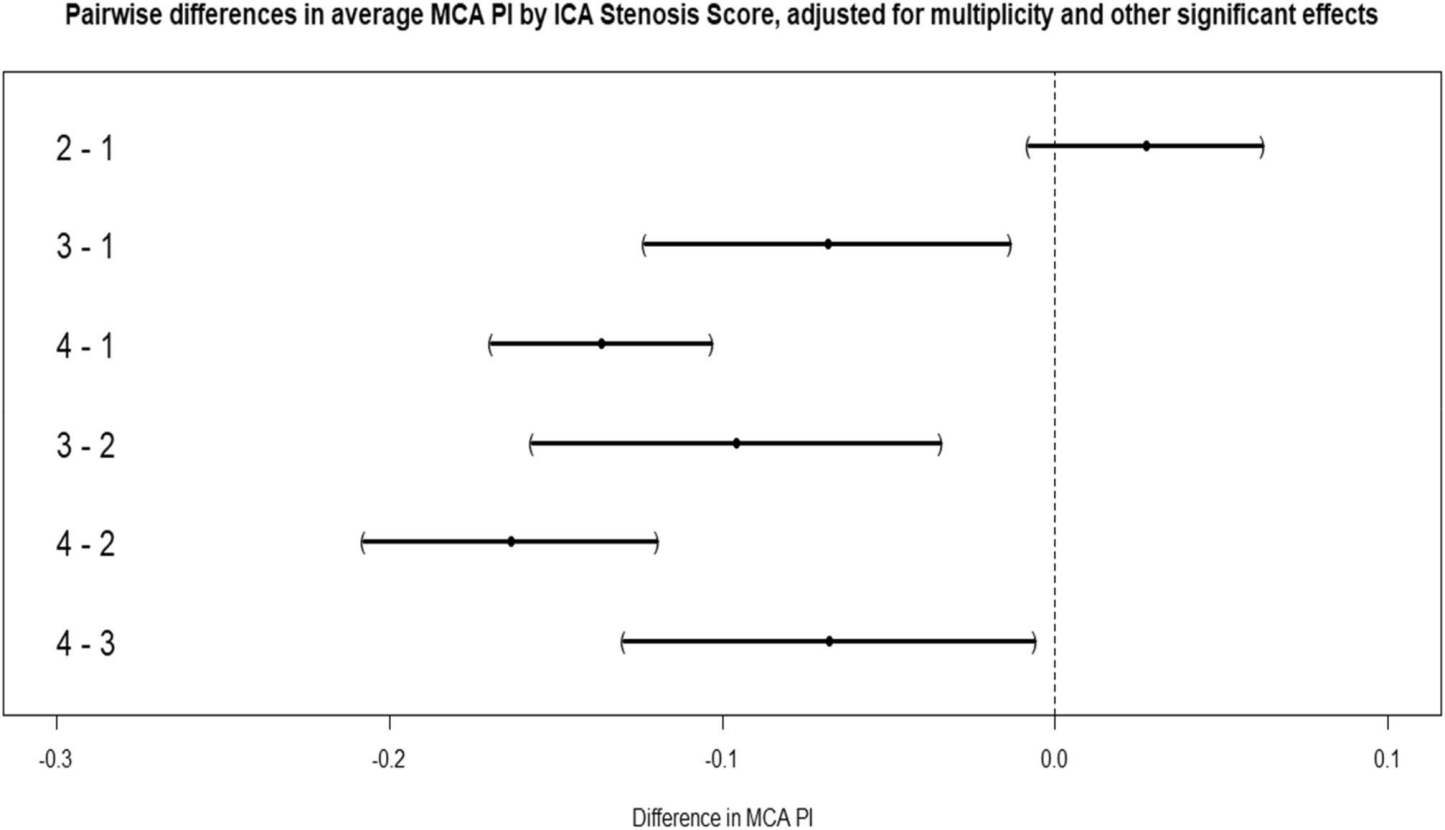
Pairwise differences according to stenosis groups, adjusted for multiplicity and other significant variables.

In a first analysis, we examined the characteristics of the patients across the 4 stenosis groups. In total, 2556 carotids had no stenosis (86.1%), the LO group (50%-70% stenosis) contained 165 carotids (5.5%), the HI group (> 70%-90%) 67 carotids (2.2%) and the SO group (> 90% or occlusion) 180 (89 R and 91L) carotids (6%). MCA PI values significantly differed by stenosis grade (*p* < 0.01). Looking at pairwise differences across groups, we observed that patients in the LO group had a trend towards higher MCA PI compared to patients with no stenosis (although this was not significant). On the contrary, patients in the HI group had significantly lower PI values than the LO or NO groups (− 0.08, 95% CI − 0.14; − 0.01). Notably, patients in the SO group had significantly lower MCA PI values than all others did (− 0.20, 95% CI − 0.25; − 0.15) (see Fig. 1). Analysing distribution of MCA PI values across age groups and sides, we found that the SO group had a lower MCA PI by 0.13 units, irrespective of side or age. In Table 1, the distribution of selected patient characteristics across stenosis groups is displayed. Women were less likely to be in the SO group (OR, 95% CI) (0.6; 0.48–0.74), as were patients with cardio-embolic stroke mechanism (0.07; CI 0.05–0.10). Among the risk factors, peripheral arterial disease was strongly associated with the SO group (2.3; 1.66–3.17) as was high NIHSS on admission (NIHSS 12 in the SO group vs NIHSS 6 to 7 in the other groups, 1.03; 1.01–1.04) and presence of a visible ischemic lesion on admission CT (1.4; 1.13–1.74). Low mRS (0–2) at 3 months (0.73; 0.59–0.91) was not strongly associated with the SO group.

**Figure 2:**
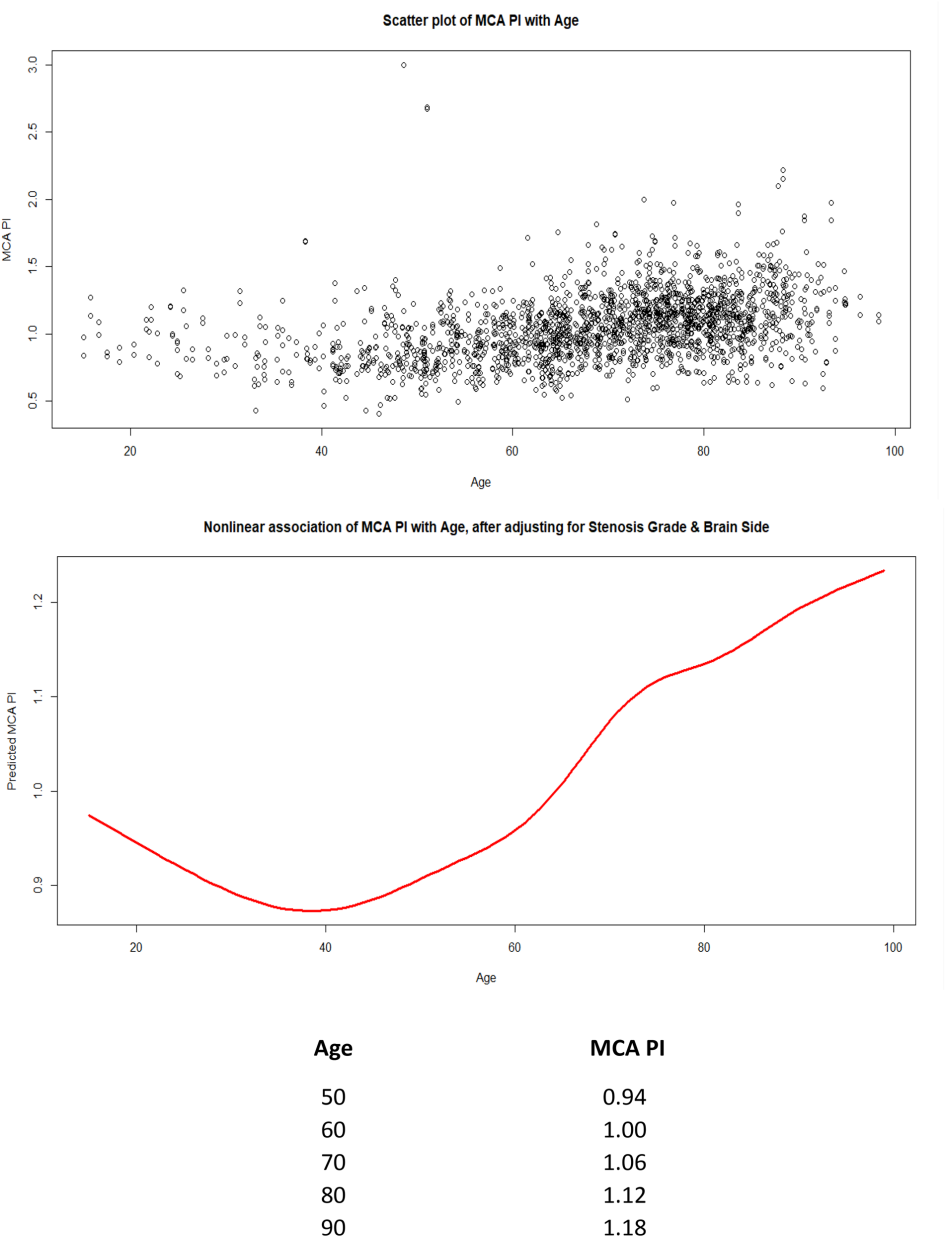
Mean MCA PI values in AIS patients without carotid artery stenosis as a function of age (scatter plot and model used mean values according to different decades).

A multivariate analysis (Table 2) showed that the following variables were independently associated with a change in MCA PI: age (Coef. 0.006; 95% CI 0.005–0.007), meaning that for each decade the MCA PI will increase by 0.006 units. Diabetes was associated with a higher MCA PI (0.071; 0.04–0.102); any acute ischemic lesion on admission CT (0.029; 0.002–0.055) as well as a severe atherosclerotic burden (0.18; 0.083–0.285). The left MCA had a reduced MCA PI by 0.0245 units compared to the right (− 0.024; 0.036–0.012). Patients in the HI group had a reduced MCA PI compared to the LO and no stenosis groups. The SO group had a reduced MCA PI compared to all other groups (− 0.068; − 0.123–0.013) and indeed, the SO group had a significant hemodynamic effect (− 0.136; − 0.136–0.103). A scatter plot of MCA PI in relation to age and a prediction model with non-linear distribution across all ages adjusted for stenosis and side are displayed in Fig. 2. The PI increases with age in a non-linear fashion and the scatter of the data is important.

**Table 2:** Multivariate analysis of significant baseline variables associated with MCA PI.

Variable	Difference in MCA PI	95% LL	95% UL	*p* value
Age (per decade)	0.006	0.005	0.007	< 1%
Diabetes	0.071	0.04	0.102	< 1%
Any ischemic lesion on admission CT	0.029	0.002	0.055	3.0%
Light atherosclerosis	− 0.013	− 0.047	0.021	45.7%
Moderate atherosclerosis	0.028	− 0.007	0.063	11.5%
Severe atherosclerosis	0.18	0.083	0.285	< 1%
Side (left vs right)	− 0.024	− 0.036	− 0.012	< 1%
LO group (50–70% stenosis)	0.027	− 0.007	0.062	12.8%
HI group (70–90% stenosis)	− 0.068	− 0.123	− 0.013	1.5%
SO group (> 90% or occlusion)	− 0.136	− 0.169	− 0.103	< 1%

### Discussion

The Pulsatility Index is an objective measure of the flow shape, independent of the insonisation angle and experience of the neurosonologist. PI has been associated with stroke outcome and recurrences^23,24^ and can be measured at low cost and without X-ray exposure. However, comprehensive, large-scale PI data in AIS patients are scarce and have only been with limited techology^25^.

This study investigated the influence of carotid artery stenosis and other patient characteristics on the PI values in the middle cerebral artery.

In a large series of AIS patients, we show that internal carotid artery stenosis > 70% is associated with lower PI values in the middle cerebral artery. Thus, when evaluating middle cerebral artery patency with transcranial Doppler, high-grade carotid stenosis should be suspected when a low MCA PI value is detected. Moreover, we show that age, diabetes, severe plaque burden, acute ischemic lesion on admission CT and right side are independently associated with higher PI values.

The fact that age and diabetes influence the pulsatility of the vessel wall, was already reported in several studies^26^ probably due to the decreased elasticity of the vessel tree in both situations. Other studies showed increases in patients with small vessel disease^27^, diabetes mellitus^28^, ageing^29,30^ and dementia^31^. A previous study showed association of increasing Resistance Index (RI) with age in lacunar stroke patients^32^. This is in line with the results from our study and may be explained by the fact that lower vessel wall pulsatility is related to its loss of elasticity^13,33–35^, which in itself is shown to be correlated to damage in the end-organs^36,37^.

Plaque ulcerations or haemorrhages have previously been shown to be of importance as they are associated with embolic signals^38^ and increased stroke risk^39,40^, even in plaques not associated with stenosis^41^. We show here that in AIS patients, plaque severity is linked to MCA PI, with an association between severe plaque morphology and increased MCA PI. The most likely explanation is that severe plaques at the carotid bifurcation are associated with small intracranial artery disease due to shared pathophysiological mechanisms. Indeed, carotid plaques were shown to be associated with lacunar strokes in the population-based Rotterdam study.

Acute ischemic lesions related to the stroke on initial CT were seen in 33.2% of the ASTRAL population^22^, which is similar to the finding in our population (41.7%). The average timing for the Doppler exam was 24 h after admission, in order to limit the hemodyamic changes related to the hyperacute phase. Early ischemic signs are seen in late window patients or early tissue and possibly microvascular damages within the acute lesion. An hypothesis could be that the presence of persisting microvascular occlusion due to pericytes swelling^42,43^ could increase vascular peripheral resistance and thus modify PI measures. Since the absence of early ischemic signs is more often seen in patients with a good collateral flow^44^, an interpretation could also be that the absence of impact on the MCA PI is due to a better collateral vasculature, whereas patients with less efficient collaterals rely more on MCA flow and thus the hemodynamic changes are observed in their case, but we did not include perfusion imaging in this study to support this point.

Pre-cerebral high-grade carotid stenosis from > 70% is significantly associated with reduced PI in the MCA distal to the stenosis or occlusion. Additionally, we find no difference in PI between normal carotid arteries (no stenosis) or low-grade stenosis. This finding is consistent with earlier work on endarterectomy patients^45–48^. Establishing clinically relevant PI cut-off values for occlusion in the MCA is unfortunately not feasible due to the large variability in mean PI seen in our patients as well as other contributing factors discussed, but also due to the possibility of spontaneous vascular status change in the hyperacute phase after an AIS^9,10^, so we suggest referring to standards per age group, or stenosis degree as described in the results.

Considering the influence of the several factors discussed on PI values and the PI variability, the MCA PI value in a given patient should be compared to the contralateral side if possible, to assess the baseline haemodynamic of the patient. A side difference would point towards a focal cause such as a sub-occlusion or occlusion rather than systemic factors. However, the left side generally shows slightly lower values (− 0.2), which is consistent with previous studies^49,50^ and could be due to the anatomical shape of the vascular tree on the left side or different flow requirements in the dominant hemisphere.

The advantages of our study are that we describe a large cohort, in a single centre with 30 years of experience in Doppler, where systematic TCD/Duplex evaluation is performed in all acute strokes, with a dedicated Doppler registry (LaDoRe); also, we limited the heterogeneity of the vascular status of patients by including only AIS and excluding chronic stroke patients. Double stenosis and pre-existing or acute intracranial stenosis were also excluded. Limitations of the study are those of a retrospective, observational, non-controlled, non-randomized study. We did not record cerebral oedema, which would increase PI, but stroke severity in our study was mild; none had a craniectomy and only a few patients had a NIHSS over 25, of whom, only four had a sub-occlusion or occlusion of the ICA. The effect of cerebral oedema on this group, if any, would tend to increase the average PI and yet we saw significantly lower PI values in this group compared to the non-stenotic group. We did not monitor cortical versus subcortical strokes, which could lead to heterogeneous results, but is consistent with common practice. We did not record details of the number of years of vascular risk factors, nor did we include radiological collateral status data, ASPECTS score^51^ or perfusion studies, which might have allowed identification of further associations or more explanations for our findings.

In conclusion, our work highlights an association of MCA PI in AIS patients with ipsilateral carotid stenosis, and age, diabetes, side of examination, ischemic lesion on admission CT and plaque morphology and provides a quantitative evaluation of the effect of these influencing factors. These aspects should be taken into account when using PI values for the TIBI^52^ score or as a surrogate marker for intracranial pressure. More studies are needed to confirm these findings and to assess the relative value of MCA PI measurement in AIS recanalization^6,7^ and reperfusion patterns.
